# Associations between health insurance status, neighborhood deprivation, and treatment delays in women with breast cancer living in Georgia

**DOI:** 10.1002/cam4.6341

**Published:** 2023-07-12

**Authors:** Sofia Awan, Geetanjali Saini, Keerthi Gogineni, Justin M. Luningham, Lindsay J. Collin, Shristi Bhattarai, Ritu Aneja, Courtney P. Williams

**Affiliations:** ^1^ School of Public Health, Georgia State University Atlanta Georgia USA; ^2^ Department of Clinical and Diagnostic Sciences, School of Health Professions University of Alabama at Birmingham Birmingham Alabama USA; ^3^ Department of Hematology–Medical Oncology Winship Cancer Institute, Emory University School of Medicine Atlanta Georgia USA; ^4^ Department of Surgery Winship Cancer Institute, Emory University School of Medicine Atlanta Georgia USA; ^5^ Georgia Cancer Center for Excellence, Grady Health System Atlanta Georgia USA; ^6^ Department of Biostatistics and Epidemiology, School of Public Health University of North Texas Health Science Center Fort Worth Texas USA; ^7^ Department of Population Health Sciences Huntsman Cancer Institute, University of Utah Salt Lake City Utah USA; ^8^ Department of Medicine, Division of Preventive Medicine University of Alabama at Birmingham Birmingham Alabama USA

**Keywords:** breast cancer, disparities, insurance coverage, socioeconomic, treatment initiation

## Abstract

**Background:**

Little is known regarding the association between insurance status and treatment delays in women with breast cancer and whether this association varies by neighborhood socioeconomic deprivation status.

**Methods:**

In this cohort study, we used medical record data of women diagnosed with breast cancer between 2004 and 2022 at two Georgia‐based healthcare systems. Treatment delay was defined as >90 days to surgery or >120 days to systemic treatment. Insurance coverage was categorized as private, Medicaid, Medicare, other public, or uninsured. Area deprivation index (ADI) was used as a proxy for neighborhood‐level socioeconomic status. Associations between delayed treatment and insurance status were analyzed using logistic regression, with an interaction term assessing effect modification by ADI.

**Results:**

Of the 14,195 women with breast cancer, 54% were non‐Hispanic Black and 52% were privately insured. Compared with privately insured patients, those who were uninsured, Medicaid enrollees, and Medicare enrollees had 79%, 75%, and 27% higher odds of delayed treatment, respectively (odds ratio [OR]: 1.79, 95% confidence interval [CI]: 1.32–2.43; OR: 1.75, 95% CI: 1.43–2.13; OR: 1.27, 95% CI: 1.06–1.51). Among patients living in low–deprivation areas, those who were uninsured, Medicaid enrollees, and Medicare enrollees had 100%, 84%, and 26% higher odds of delayed treatment than privately insured patients (OR: 2.00, 95% CI: 1.44–2.78; OR: 1.84, 95% CI: 1.48–2.30; OR: 1.26, 95% CI: 1.05–1.53). No differences in the odds of delayed treatment by insurance status were observed in patients living in high‐deprivation areas.

**Discussion/Conclusion:**

Insurance status was associated with treatment delays for women living in low‐deprivation neighborhoods. However, for women living in neighborhoods with high deprivation, treatment delays were observed regardless of insurance status.

## Graphical abstract

This study of women with breast cancer living in Georgia showed that patients who were Medicare enrollees, Medicaid enrollees, and uninsured had significantly higher odds of treatment delays compared to privately insured patients. Significant differences in odds of treatment delays by insurance coverage were observed for patients living in neighborhoods of low deprivation but not for those living in areas of high deprivation. Policy and interventions should target equity in insurance coverage to improve timely treatment initiation.

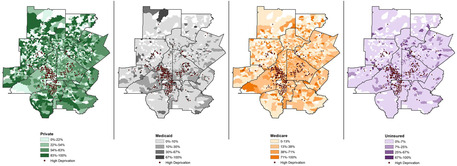

## 1 INTRODUCTION

Delays in breast cancer treatment initiation are associated with adverse cancer outcomes, such as incomplete or forgone treatment; more intensive, frequent, or long‐term treatment; cancer progression; treatment complications; lessened quality of life; and decreased survival.^1^, ^2^, ^3^, ^4^, ^5^, ^6^ Increases in insurance coverage, such as through the Patient Protection and Affordable Care Act (ACA), have facilitated increases in healthcare access. Therefore, insurance coverage may decrease treatment delays by reducing potential cost‐related barriers to care.^7^ However, insurance coverage itself may not mitigate all cancer‐related financial burdens. Although the number of individuals without insurance decreased after the passage of the ACA, the number of underinsured individuals or individuals who spend >10% of their household income on out‐of‐pocket medical costs continues to rise.^8^, ^9^ Furthermore, insurance coverage cannot offset indirect breast cancer‐related expenses, such as transportation to care, childcare while receiving care, and reduced income from time off work to receive treatment.

Due to the direct and indirect breast cancer care costs not covered by insurance, it is necessary to consider patients' socioeconomic position when analyzing potential care access barriers.^10^, ^11^, ^12^ Previous studies have shown that individuals with cancer residing in disadvantaged neighborhoods (e.g., residents with low household income, low education levels, high unemployment rate, and low homeownership rate) have higher overall and breast cancer‐specific mortality,^13^, ^14^ higher breast cancer incidence,^15^ and lower healthcare access^16^ than those living in neighborhoods of less disadvantage. Care access barriers may also be greater for women with breast cancer living in the state of Georgia, where rates of uninsurance and poverty are higher than those in other areas of the United States.^17^, ^18^, ^19^

In this study, we assessed the relationship between insurance status and treatment delays in women diagnosed with breast cancer living in Georgia. Additionally, we explored how this association may differ for individuals living in neighborhoods of varying socioeconomic disadvantage.

## 2 METHODS

### 2.1 Study design and sample

**FIGURE 1:**
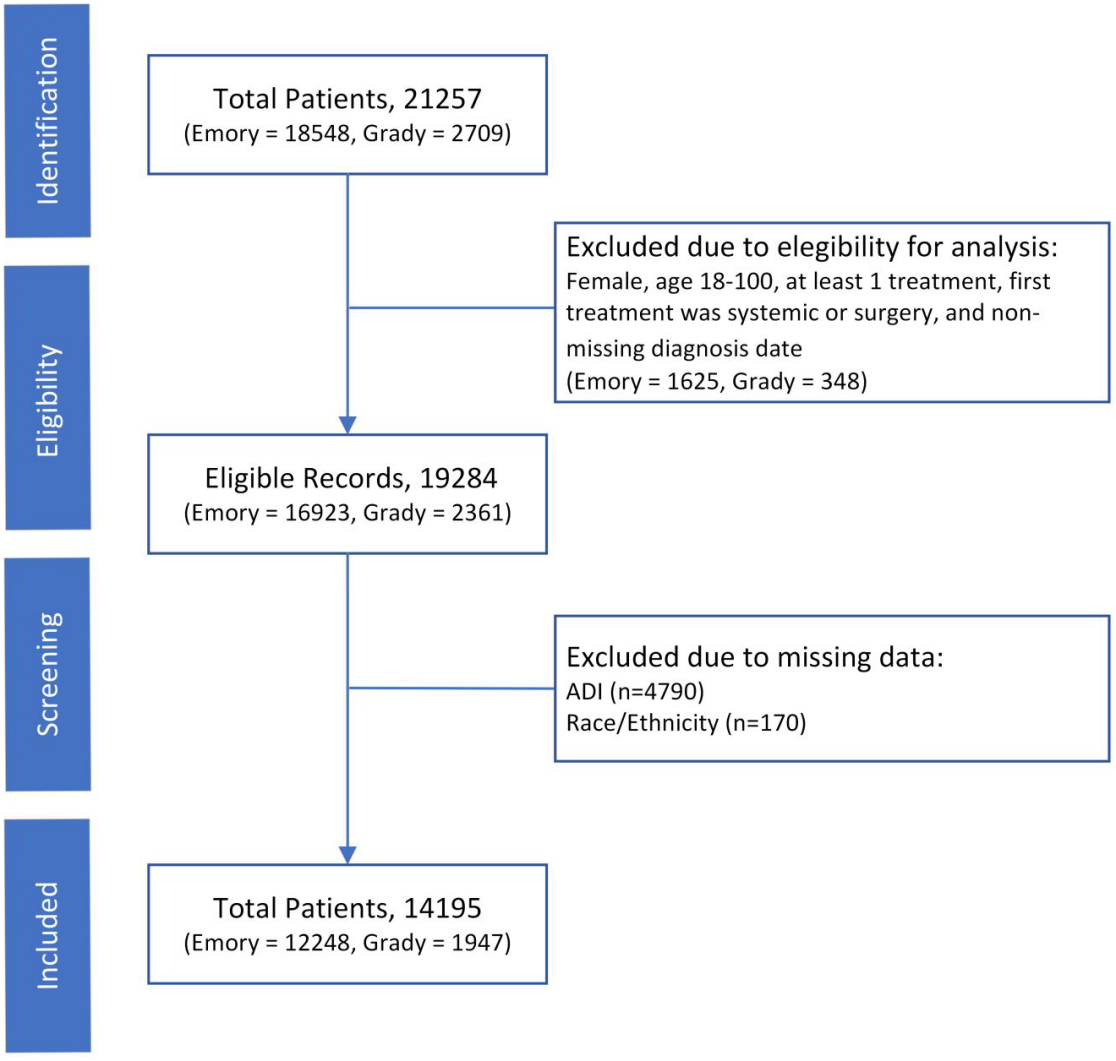
Study sample exclusion cascade.

This retrospective cohort study included women with breast cancer receiving care at either of two large, academic, public and private, Georgia‐based health care systems, Grady Health System and Emory Healthcare. Women ≥18 years of age diagnosed with breast cancer between 2004 and 2022 and who received at least one treatment for breast cancer were included (Figure 1). Data for this study were abstracted from electronic patient records by hospital administrators. Patients with missing data for age, race, ethnicity, treatment start dates, and address or nine‐digit zip codes were excluded. Unknown categories were created for American Joint Committee on Cancer (AJCC) stage and breast cancer subtype variables to address high rates of missing data for these variables. This study was approved by the Georgia State University Institutional Review Board (IRB) and by the hospitals providing data. The IRB waived the need for consent as medical record data were collected retrospectively.

### 2.2 Delayed treatment

The outcome for our study was delayed treatment. Patients were considered to have received delayed treatment if the number of days from breast cancer diagnosis to initiation of breast cancer treatment was higher than that recommended in prior studies and the National Accreditation Program for Breast Centers guidelines.^6^, ^20^, ^21^ Breast cancer treatments included surgery, neoadjuvant systemic therapy, and other (systemic therapy only, radiation therapy only, systemic and radiation therapy). Surgical treatment refers to time from diagnosis to curative surgery date, and does not include excisional biopsies or non‐cancer directed surgeries. Delayed treatment was defined as treatment initiated >90 days after diagnosis for patients receiving surgery as first treatment and >120 days for those receiving systemic therapy first (i.e., chemotherapy, endocrine therapy, and targeted therapy).

### 2.3 Exposure: Insurance coverage status

Health insurance coverage was captured during the initial oncology clinic visit and classified into one of the following six categories: private insurance, Medicaid, Medicare, other public coverage (Indian Health Service, Military, and Veterans Affairs), none (i.e., uninsured), and unknown.

### 2.4 Neighborhood deprivation

Neighborhood deprivation was used as a proxy for patient socioeconomic status and was calculated using the Area deprivation index (ADI).^22^ The ADI is a composite measure comprised of 17 metrics of income, education, employment, and housing quality determined using census block group 12‐digit Federal Information Processing Standards (FIPS) codes or nine‐digit zip codes. ADI scores were calculated based on the patient's home address at breast cancer diagnosis by geocoding the address to a 12‐digit FIPS code using the United States Census Bureau's Census Geocoder^23^ and to a nine‐digit zip code using the US Postal Service zip code database.^24^ Nine‐digit zip codes or 12‐digit FIPS codes were matched to census block ADI scores obtained from the Neighborhood Atlas website.^25^, ^26^ The ADI is scored from 1 to 100, with higher scores indicating greater neighborhood deprivation. Areas with ADI scores ≥85 were considered neighborhoods of high deprivation, whereas those with scores <85 were considered neighborhoods of low deprivation. These categories of ADI scores were based on previous studies using similar cut points.^27^, ^28^, ^29^

### 2.5 Covariates

Abstracted patient demographic information included age at diagnosis, hospital, and race and ethnicity (non‐Hispanic White, non‐Hispanic Black, Hispanic, and other race). Abstracted patient clinicopathologic data included AJCC stage (0, I, II, III, IV), breast cancer subtype (hormone receptor [HR]+/human epidermal growth factor receptor 2 [HER2]+, HR−/HER2−, HR+/HER2−, HR−/HER2+), and treatment modality (neoadjuvant [systemic treatment followed by surgery], surgery [surgery as first treatment], and other [radiation as first treatment or systemic treatment only]).

### 2.6 Statistical analysis

Descriptive statistics were calculated using frequency counts and percentages for categorical variables and medians and interquartile ranges (IQR) for continuous variables. The association of delayed treatment and insurance status was estimated using odds ratios (OR) and 95% confidence intervals (CI) using a logistic regression model. The model was adjusted for patient age at diagnosis, race and ethnicity, hospital, neighborhood deprivation, treatment modality, stage, and breast cancer subtype. To understand the effect of neighborhood deprivation on the relationship between time to treatment initiation and insurance status, an interaction term was added to our model. Interaction significance was assessed using likelihood ratio tests, which did not result in a significantly different fit. Multicollinearity of model covariates was evaluated using the “car” package^30^ in R, which estimates variance inflation factors. Absence of multicollinearity was confirmed for all covariates included in the final logistic regression model. Data cleaning, analysis, and modeling were performed using R version 4.2 (Vienna, Austria) and SAS software version 9.4.^31^ To qualitatively compare geographic clusters of deprivation by insurance status, we created maps using ArcGIS and US Census Bureau shapefiles.^32^, ^33^

## 3 RESULTS

### 3.1 Sample characteristics

Data from 14,195 of the 19,284 (74%) women with breast cancer in our dataset were included in our analysis (Figure 1) after excluding patients with missing data for age, race, ethnicity, and ADI score. Compared to included patients, excluded patients were more often Medicare enrolled (36% vs. 28%) and were less often non‐Hispanic Black (35% vs. 54%) and living at low neighborhood deprivation (5% vs. 85%; Supplemental Table S1). Among the included patients, median age at diagnosis was 58 years (IQR 49–67) and 54% of patients were non‐Hispanic Black. Patients were most often diagnosed with stage I disease (30%), 37% had HR+/HER2− breast cancer, and 74% underwent surgery as their first treatment.

Over half of the included patients were privately insured (52%), 28% were Medicare enrollees, 11% were Medicaid enrollees, 4% were uninsured, and <1% had other public insurance (Table 1). The majority of privately insured patients were non‐Hispanic White (49%) or non‐Hispanic Black (49%). Non‐Hispanic Black patients were more often Medicaid enrollees (17% vs. 4%) or uninsured (5% vs. 2%), compared to non‐Hispanic White patients.

**TABLE 1:** Patient demographic and clinicopathological characteristics (*N* = 14,195).

	Total	Private	Medicaid	Medicare	Uninsured	Other public	Unknown
	14,195 (100)	7348 (52)	1610 (11)	3927 (28)	616 (4)	63 (<1)	631 (4)
Demographics							
Race							
NHB	7706 (54)	3611 (49)	1293 (80)	2014 (51)	401 (65)	43 (68)	344 (55)
NHW	6138 (43)	3627 (49)	248 (15)	1878 (48)	97 (16)	20 (32)	268 (42)
Hispanic	307 (2)	110 (1)	51 (3)	34 (1)	98 (16)	0 (0)	14 (2)
Other	44 (<1)	0 (0)	18 (1)	1 (<1)	20 (3)	0 (0)	5 (1)
ADI							
High deprivation ≥85	2148 (15)	733 (10)	467 (29)	668 (17)	151 (25)	14 (22)	115 (18)
Low deprivation<85	12,047 (85)	6615 (90)	1143 (71)	3259 (83)	465 (75)	49 (78)	516 (82)
Characteristics							
Age at diagnosis	58 (49–67)	54 (46–60)	52 (44–59)	71 (66–76)	52 (45–60)	59 (50–63)	56 (48–64)
TTI (days)	40 (24–61)	40 (23–60)	44 (28–68)	39 (24–60)	43 (28–69)	54 (34–97)	35 (18–53)
Clinicopathological factors							
First treatment							
Surgery	10,444 (74)	5531 (75)	892 (55)	3145 (80)	383 (62)	36 (57)	457 (72)
Systemic	3751 (26)	1817 (25)	718 (45)	782 (20)	233 (38)	27 (43)	174 (28)
Treatment modality							
Neoadjuvant therapy	2784 (20)	1505 (20)	511 (32)	480 (12)	152 (25)	18 (29)	118 (19)
Surgery	10,444 (74)	5531 (75)	892 (55)	3145 (80)	383 (62)	36 (57)	457 (72)
Other	967 (7)	312 (4)	207 (13)	302 (8)	81 (13)	9 (14)	56 (9)
Stage							
0	2175 (15)	1225 (17)	196 (12)	542 (14)	96 (16)	7 (11)	109 (17)
I	4252 (30)	2184 (30)	346 (21)	1362 (35)	138 (22)	20 (32)	202 (32)
II	3316 (23)	1732 (24)	456 (28)	834 (21)	115 (19)	13 (21)	166 (26)
III	454 (3)	165 (2)	111 (7)	108 (3)	45 (7)	1 (2)	24 (4)
IV	222 (2)	91 (1)	50 (3)	50 (1)	16 (3)	2 (3)	13 (2)
Unknown	3776 (27)	1951 (27)	451 (28)	1031 (26)	206 (33)	20 (32)	117 (19)
Hormone type							
HR+/HER2+	922 (6)	493 (7)	124 (8)	228 (6)	43 (7)	7 (11)	27 (4)
HR−/HER2−	1313 (9)	610 (8)	205 (13)	349 (9)	87 (14)	9 (14)	53 (8)
HR−/HER2+	430 (3)	213 (3)	75 (5)	108 (3)	16 (3)	6 (10)	12 (2)
HR+/HER2−	5192 (37)	2518 (34)	531 (33)	1792 (46)	189 (31)	24 (38)	138 (22)
Unknown	6338 (45)	3514 (48)	675 (42)	1450 (37)	281 (46)	17 (27)	401 (64)

*Note*: Values are expressed as *n* (%) or median (IQR).

Abbreviations: IQR, interquartile range; NHB, non‐Hispanic Black; NHW, non‐Hispanic White; TTI, time to treatment initiation.

### 3.2 Delayed treatment and insurance status

Median time to treatment initiation was 43 days for uninsured patients (IQR 28–69), 44 days for Medicaid enrollees (IQR 28–68), 40 days for privately insured patients (IQR 23–60), and 39 days for Medicare enrollees (IQR 24–60; Table 1). A total of 9% (n = 1241) of patients received delayed treatment, which varied by first treatment type. Of patients who underwent surgery as first treatment, 11% experienced delays. Medicaid enrollees and patients with other public insurance had the longest time to surgical treatment initiation (median 45 days, IQR 27–70 and median 57 days, IQR 35–108, respectively; Table 2). Only 4% of patients who received systemic therapy first received delayed treatment. Patients who were uninsured had the longest time to systemic treatment initiation (median 47 days, IQR 30–66; Table 2).

**TABLE 2:** Time to first treatment initiation by insurance coverage status (*N* = 14,195).

	Private	Medicaid	Medicare	Uninsured	Other public	Unknown
Surgery	42 (24–62)	45 (27–70)	40 (25–61)	42 (26–70)	57 (35–108)	32 (15–52)
Systemic	35 (23–51)	43 (29–64)	37 (23–56)	47 (30–66)	38 (34–73)	39 (26–55)

*Note*: Values are expressed as median (IQR).

Abbreviation: IQR, interquartile range.

In our multivariable‐adjusted model, uninsured patients had 79% higher odds of experiencing treatment delays compared to those privately insured (OR: 1.79, 95% CI: 1.32–2.43; Table 3). Compared to patients with private insurance, Medicaid enrollees had 75% higher odds and Medicare enrollees 27% higher odds of experiencing treatment delays (OR: 1.75, 95% CI: 1.43–2.13; OR: 1.27, 95% CI: 1.06–1.51, respectively).

**TABLE 3:** Multivariable model‐estimated odds of delayed treatment by insurance status (non‐interaction model) and insurance status modified by neighborhood deprivation (interaction model; *N* = 14,195).^a^

	Non‐interaction model OR (95% CI)	Interaction model OR (95% CI)	
		High neighborhood deprivation	Low neighborhood deprivation
Private	1 [Reference]	1 [Reference]	1 [Reference]
Medicaid	1.75 (1.43–2.13)	1.46 (0.98–2.20)	1.84 (1.48–2.30)
Medicare	1.27 (1.06–1.51)	1.26 (0.87–1.81)	1.26 (1.05–1.53)
Uninsured	1.79 (1.32–2.43)	1.20 (0.63–2.29)	2.00 (1.44–2.78)
Other public	4.42 (2.38–8.20)	2.82 (0.73–10.87)	5.01 (2.50–10.03)
Unknown	0.99 (0.71–1.37)	1.18 (0.58–2.40)	0.94 (0.65–1.35)

^a^ Models adjusted for age at diagnosis, hospital, race and ethnicity, neighborhood deprivation, AJCC stage, breast cancer subtype, and treatment modality.

Abbreviations: CI, confidence interval; OR, odds ratio.

### 3.3 Modification by neighborhood deprivation

Patients living in neighborhoods of high deprivation comprised 15% of our sample. Compared with those living in neighborhoods of low deprivation, patients living in neighborhoods of high deprivation were more often non‐Hispanic Black (85% vs. 49%). Women living in neighborhoods of high versus low deprivation were less often privately insured (34% vs. 55%) and were more often enrolled in Medicaid, Medicare, or uninsured (22% vs. 9%, 31% vs. 27%, and 7% vs. 4%, respectively). Patients living in neighborhoods of higher vs. lower deprivation also had similar median time to treatment initiation (43 days, IQR 27–65 vs. 40 days, IQR 24–60).

**FIGURE 2:**
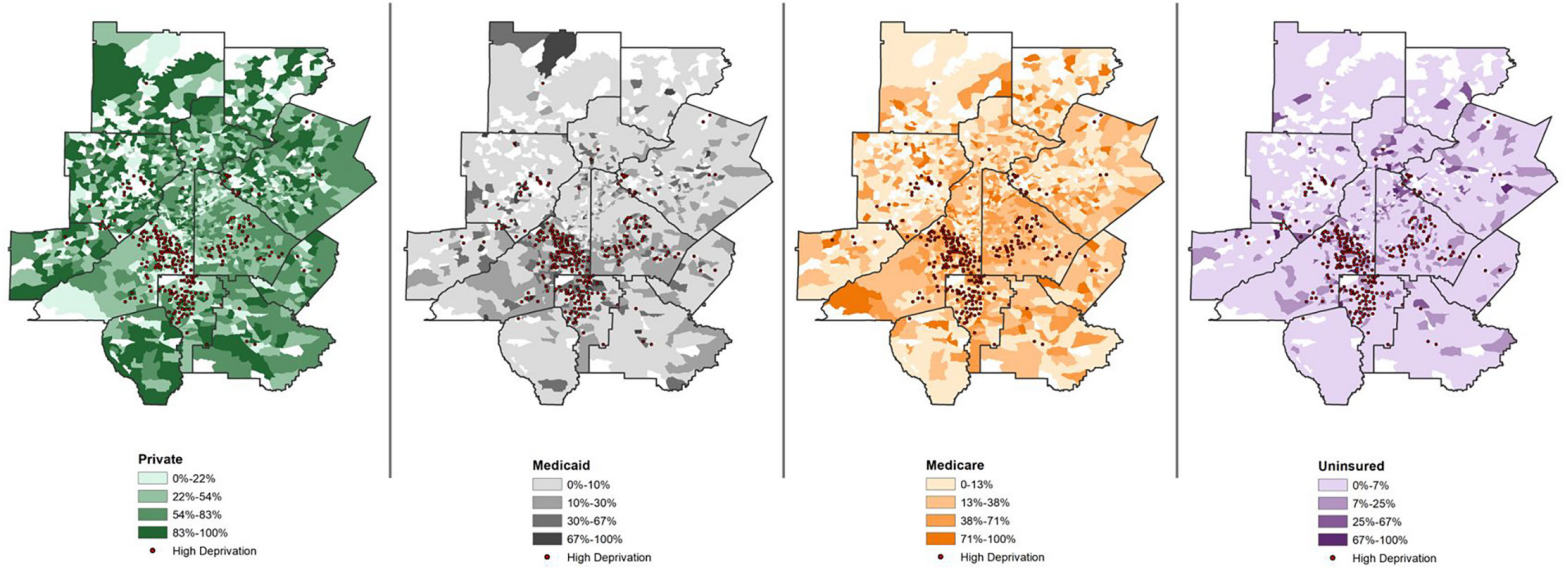
Patient census block group area deprivation and insurance status. Block groups of high deprivation contrasted across the percentage of patients in each block group with insurance coverage of private, Medicaid, Medicare, or uninsured. Red dots symbolize block groups of high neighborhood deprivation.

In qualitative geographical comparisons, we observed higher concentrations of patients who were Medicaid or Medicare enrollees living in areas of higher deprivation compared to patients with private insurance (Figure 2). For patients living in neighborhoods of high deprivation, the odds of delayed treatment did not differ between patients covered by different insurance (Table 3). Conversely, patients living in low‐deprivation neighborhoods who were uninsured, Medicaid beneficiaries, or Medicare beneficiaries had 100%, 84%, and 26% higher odds of delayed treatment compared with privately insured patients (OR: 2.00, 95% CI: 1.44–2.78; OR: 1.84, 95% CI: 1.48–2.30; OR: 1.26, 95% CI: 1.05–1.53, respectively).

## 4 DISCUSSION

In this study of women with breast cancer living in Georgia, we found that patients who were Medicare enrollees, Medicaid enrollees, and uninsured had higher odds of treatment delays than privately insured patients. Furthermore, the odds of treatment delays by insurance coverage differed for patients living in neighborhoods of low and high deprivation, with no differences in treatment delays found for patients living in areas of high deprivation. Our findings are consistent with prior research on treatment delays by insurance status^34^ and provide additional support for the role health insurance plays in lessening health inequities in Georgia by decreasing barriers to cancer care access.

Compared with privately insured patients, patients who were uninsured had 79% higher odds of delayed treatment. Studies have shown that lacking insurance is associated with reduced breast cancer screening, later stage at diagnosis, and decreased survival.^35^, ^36^ Previous studies have found that ACA Medicaid expansion increased insurance coverage for uninsured individuals.^37^, ^38^ Additionally, Medicaid expansions have been shown to reduce racial health disparities by improving health coverage and thus improving health outcomes.^39^, ^40^ As of 2023, 11 states, including Georgia, have not adopted Medicaid expansion.^41^ Medicaid expansions are an avenue that could alleviate healthcare disparities and distinctly impact cancer care access barriers in Georgia.

Patients who were publicly insured through Medicaid or Medicare also faced higher odds of treatment delays compared to privately insured patients. We found that compared to patients with private insurance, those who were Medicare insured had 27% higher odds of delayed treatment. Treatment delays seen in patients with Medicare coverage may be due to age related factors, as older age was a patient‐related factor shown to increase time to treatment.^42^ A study found moderate delays in breast cancer treatment to occur more frequently in older patients, however longer delays were not common.^43^ Compared to patients who were privately insured, Medicaid enrollees had 75% higher odds of delayed treatment, higher than the odds for Medicare enrollees. Studies have shown that Medicaid coverage is less costly to patients than private insurance, but a mixed association exists between care quality and insurance coverage.^44^ However, Medicaid enrollees may face more barriers to care due to indirect care costs uncovered by insurance, since lower socioeconomic status has been associated with increased treatment delays.^45^, ^46^ Thus, there appears to be a need for interventions that target inequities due to both variation in insurance coverage and underlying sociodemographic factors.

For patients living in low‐deprivation neighborhoods, the odds of treatment delays differed by insurance status, with privately insured women less likely to experience delays than all other groups; however, patients residing in high‐deprivation neighborhoods were equally as likely to experience treatment delays across all insurance types. These results suggest that insurance coverage alone is inadequate in accounting for cost‐related challenges associated with access to breast cancer treatment. The ability to receive timely care is also affected by socioeconomic factors, such as transportation, income protection (sick leave), and housing stability. However, the extent to which geographical deprivation impacts delayed treatment for different insurance types is unclear. While area deprivation measures have been shown to be predictive of cancer treatment outcomes,^47^, ^48^ it does not provide a comprehensive measure of socioeconomic challenges. Future research is needed on the social determinants of health with respect to residence and breast cancer treatment delays to inform policy and intervention strategies aimed at improving health equity.

### 4.1 Limitations

Results from this study should be considered within the context of several limitations. This study assumes that patients did not receive care at other facilities and that treatment delays were not due to patient requests, such as a patient's decision to receive a second opinion on a treatment plan at another hospital. The address was available only for residence at the time of diagnosis. ADI provides information on neighborhood deprivation rather than individual‐level socioeconomic status. We also did not have information for other factors affecting access to care due to residence, such as availability of public transportation, walkability, and safety. Few patients were covered by non‐Medicare or Medicaid public insurance, which limited our ability to reliably compare associations for this group. Furthermore, this study was limited in the geographic generalizability of results because most patients were located and treated in the Atlanta metropolitan area.

## 5 CONCLUSION

This study of women with breast cancer living in Georgia showed that patients who were Medicare enrollees, Medicaid enrollees, and uninsured had significantly higher odds of treatment delays compared to privately insured patients. Furthermore, significant differences in odds of treatment delays by insurance coverage were observed for patients living in neighborhoods of low deprivation but not for those living in areas of high deprivation. Policy and interventions should target equity in insurance coverage to improve timely treatment initiation. Additional studies may help to identify strategies to mitigate treatment delays for patients living in areas of high deprivation.

## AUTHOR CONTRIBUTIONS

**Sofia Awan:** Conceptualization (equal); data curation (equal); formal analysis (lead); methodology (equal); visualization (lead); writing – original draft (lead); writing – review and editing (equal). **Geetanjali Saini:** Conceptualization (equal); data curation (equal); writing – review and editing (equal). **keerthi gogineni:** Conceptualization (equal); methodology (equal); writing – review and editing (equal). **Justin M. Luningham:** Conceptualization (equal); data curation (equal); methodology (equal); writing – review and editing (equal). **Lindsay J. Collin:** Conceptualization (equal); methodology (equal); writing – review and editing (equal). **Shristi Bhattarai:** Data curation (equal); writing – review and editing (equal). **Ritu Aneja:** Conceptualization (equal); data curation (equal); funding acquisition (equal); supervision (equal); writing – review and editing (equal). **Courtney P Williams:** Conceptualization (equal); formal analysis (equal); methodology (equal); supervision (equal); writing – original draft (equal); writing – review and editing (equal).

## FUNDING INFORMATION

This study was supported by grant R01CA239120 from the National Cancer Institute (Dr Aneja). Lindsay J. Collin was supported by K99CA277580 from the National Cancer Institute of the National Institutes of Health.

## CONFLICT OF INTEREST STATEMENT

No conflicts of interest to declare.

## Supporting information


Table S1.

## Data Availability

Data used for this study is available from the corresponding authors upon reasonable request.
