# Supplementary material for: Associations between health insurance status, neighborhood deprivation, and treatment delays in women with breast cancer living in Georgia
Source: Cancer Med. 2023 Jul 12;12(16):17331–9. doi: 10.1002/cam4.6341 (PMC10501236; doi:10.1002/cam4.6341)
Supplement: Supplementary file 1 — Table S1. [file CAM4-12-17331-s001.docx]

**Supplemental Figures and Tables**

| **Supplemental Table 1.** Excluded patients' demographic and clinicopathological characteristics (N=5,089) | | | | | | | | | | | | | | | |
| --- | --- | --- | --- | --- | --- | --- | --- | --- | --- | --- | --- | --- | --- | --- | --- |
|  | **Total** | | **Private** | | **Medicaid** | | **Medicare** | | **Uninsured** | | **Other Public** | | **Unknown** | |  |
|  | **N** | **%** | **N** | **%** | **N** | **%** | **N** | **%** | **N** | **%** | **N** | **%** | **N** | **%** |  |
|  | 5089 | 100% | 2201 | 43% | 390 | 8% | 1820 | 36% | 215 | 4% | 46 | 1% | 417 | 8% |  |
| **Demographics** |  |  |  |  |  |  |  |  |  |  |  |  |  |  |  |
| **Race** |  |  |  |  |  |  |  |  |  |  |  |  |  |  |  |
| NHB | 1778 | 35% | 608 | 28% | 209 | 54% | 706 | 39% | 91 | 42% | 25 | 54% | 139 | 33% |  |
| NHW | 1756 | 35% | 727 | 33% | 44 | 11% | 751 | 41% | 25 | 12% | 10 | 22% | 199 | 48% |  |
| Hispanic | 84 | 2% | 28 | 1% | 10 | 3% | 14 | 1% | 22 | 10% | 1 | 2% | 9 | 2% |  |
| Other | 700 | 14% | 419 | 19% | 79 | 20% | 129 | 7% | 44 | 20% | 3 | 7% | 26 | 6% |  |
| NA | 771 | 15% | 419 | 19% | 48 | 12% | 220 | 12% | 33 | 15% | 7 | 15% | 44 | 11% |  |
| **ADI** |  |  |  |  |  |  |  |  |  |  |  |  |  |  |  |
| High deprivation ≥85 | 34 | 1% | 10 | 0% | 6 | 2% | 14 | 1% | 2 | 1% | 0 | 0% | 2 | 0% |  |
| Low deprivation<85 | 265 | 5% | 152 | 7% | 16 | 4% | 73 | 4% | 10 | 5% | 2 | 4% | 12 | 3% |  |
| NA | 4790 | 94% | 2039 | 93% | 368 | 94% | 1733 | 95% | 203 | 94% | 44 | 96% | 403 | 97% |  |
| **Characteristics** |  |  |  |  |  |  |  |  |  |  |  |  |  |  |  |
|  | **Median** | **IQR** | **Median** | **IQR** | **Median** | **IQR** | **Median** | **IQR** | **Median** | **IQR** | **Median** | **IQR** | **Median** | **IQR** |  |
| Age at diagnosis | 59 | 49-69 | 53 | 45-59 | 50 | 42-58 | 71 | 66-77 | 53 | 46-60 | 50 | 44-56 | 56 | 48-65 |  |
| TTI (days) | 37 | 22-57 | 37 | 22-56 | 42 | 27-63 | 38 | 22-58 | 43 | 27-64 | 47 | 35-82 | 27 | 13-49 |  |
| **Clinicopathological factors** |  |  |  |  |  |  |  |  |  |  |  |  |  |  |  |
| **1st Treatment** | **N** | **%** | **N** | **%** | **N** | **%** | **N** | **%** | **N** | **%** | **N** | **%** | **N** | **%** |  |
| Surgery | 3692 | 73% | 1616 | 73% | 212 | 54% | 1374 | 75% | 122 | 57% | 35 | 76% | 333 | 80% |  |
| Systemic | 1397 | 27% | 585 | 27% | 178 | 46% | 446 | 25% | 93 | 43% | 11 | 24% | 84 | 20% |  |
| **Treatment modality** |  |  |  |  |  |  |  |  |  |  |  |  |  |  |  |
| Neoadjuvant therapy | 916 | 18% | 442 | 20% | 132 | 34% | 206 | 11% | 70 | 33% | 10 | 22% | 56 | 13% |  |
| Surgery | 3692 | 73% | 1616 | 73% | 212 | 54% | 1374 | 75% | 122 | 57% | 35 | 76% | 333 | 80% |  |
| Other | 481 | 9% | 143 | 6% | 46 | 12% | 240 | 13% | 23 | 11% | 1 | 2% | 28 | 7% |  |
| **Stage** |  |  |  |  |  |  |  |  |  |  |  |  |  |  |  |
| 0 | 758 | 15% | 356 | 16% | 32 | 8% | 262 | 14% | 24 | 11% | 10 | 22% | 74 | 18% |  |
| I | 1436 | 28% | 653 | 30% | 91 | 23% | 478 | 26% | 58 | 27% | 11 | 24% | 145 | 35% |  |
| II | 1005 | 20% | 480 | 22% | 109 | 28% | 288 | 16% | 36 | 17% | 9 | 20% | 83 | 20% |  |
| III | 108 | 2% | 36 | 2% | 18 | 5% | 37 | 2% | 11 | 5% | 0 | 0% | 6 | 1% |  |
| IV | 84 | 2% | 28 | 1% | 14 | 4% | 30 | 2% | 4 | 2% | 0 | 0% | 8 | 2% |  |
| Unknown | 1698 | 33% | 648 | 29% | 126 | 32% | 725 | 40% | 82 | 38% | 16 | 35% | 101 | 24% |  |
| **Hormone type** |  |  |  |  |  |  |  |  |  |  |  |  |  |  |  |
| ER+/PR+/HER2+ | 293 | 6% | 148 | 7% | 26 | 7% | 90 | 5% | 16 | 7% | 3 | 7% | 10 | 2% |  |
| ER-/PR-/HER2- | 409 | 8% | 146 | 7% | 58 | 15% | 133 | 7% | 38 | 18% | 2 | 4% | 32 | 8% |  |
| ER-/PR-/HER2+ | 150 | 3% | 75 | 3% | 22 | 6% | 42 | 2% | 6 | 3% | 1 | 2% | 4 | 1% |  |
| ER+/PR+/HER2- | 1576 | 31% | 681 | 31% | 123 | 32% | 629 | 35% | 68 | 32% | 9 | 20% | 66 | 16% |  |
| Unknown | 2661 | 52% | 1151 | 52% | 161 | 41% | 926 | 51% | 87 | 40% | 31 | 67% | 305 | 73% |  |

IQR: interquartile range; NHB: non-Hispanic Black; NHW: non-Hispanic White; TTI: time to treatment initiation.
